# Extracellular Vesicles as Drivers of Non-Alcoholic Fatty Liver Disease: Small Particles with Big Impact

**DOI:** 10.3390/biomedicines9010093

**Published:** 2021-01-19

**Authors:** David Højland Ipsen, Pernille Tveden-Nyborg

**Affiliations:** Department of Veterinary and Animal Sciences, Section of Experimental Animal Model, University of Copenhagen, Ridebanevej 9, 1870 Frederiksberg, Denmark; dhi@sund.ku.dk

**Keywords:** nonalcoholic fatty liver disease, nonalcoholic steatohepatitis, extracellular vesicles

## Abstract

Nonalcoholic fatty liver disease (NAFLD) is becoming the leading chronic liver disease, negatively affecting the lives of millions of patients worldwide. The complex pathogenesis involves crosstalk between multiple cellular networks, but how the intricate communication between these cells drives disease progression remains to be further elucidated. Furthermore, the disease is not limited to the liver and includes the reprogramming of distant cell populations in different organs. Extracellular vesicles (EVs) have gained increased attention as mediators of cellular communication. EVs carry specific cargos that can act as disease-specific signals both locally and systemically. Focusing on NAFLD advancing to steatohepatitis (NASH), this review provides an update on current experimental and clinical findings of the potential role of EVs in hepatic inflammation and fibrosis, the main contributors to progressive NASH. Particular attention is placed on the characteristics of EV cargos and potential specificity to disease stages, with putative value as disease markers and treatment targets for future investigations.

## 1. Introduction

**Figure 1:**
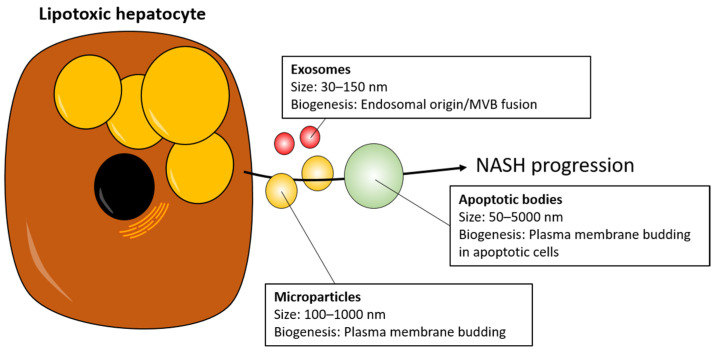
Lipotoxic hepatocytes release extracellular vesicles (EVs). Lipotoxic hepatocytes release EVs of various sizes and origins that can be subdivided into exosomes, microparticles, and apoptotic bodies. Exosome biogenesis is initiated by the inward budding of the endosomal membrane resulting in the formation of MVBs. These MVBs can then fuse with the plasma membrane, which releases the exosomes into the extracellular space. Both microparticles and apoptotic bodies result from the direct outward budding of the plasma membrane, with the latter from apoptotic cells [6]. MVB: multivesicular bodies. NASH: nonalcoholic steatohepatitis. Large, yellow circles: Intracellular lipid vesicles in hepatocyte; Black circle: Hepatocyte nucleus.

Nonalcoholic fatty liver disease (NAFLD) affects 25% of the world’s population and encompasses a spectrum of hepatic conditions ranging from hepatic steatosis (termed NAFL) to inflammation (NASH), which can progress to fibrosis and ultimately cirrhosis [1]. Perplexingly, while considered a progressing disease, only up to 30% of NAFLD patients develop NASH, and it remains unclear what factors cause some patients to progress while others do not [1]. Hepatic lipid levels are increased in the early disease stages and are linked to the pathogenesis of the disease. Lipids such as free fatty acids, free cholesterol, diacylglycerols, ceramides, and phospholipids accumulate in hepatocytes with cell-damaging effects through lipotoxicity [2]. These lipotoxic hepatocytes are then capable of triggering and sustaining an inflammatory signaling cascade, proposedly through the release of extracellular vesicles (EVs) [2]. EVs are a small heterogeneous collection of particles released by cells and are characterized into three broad categories based on their size and biogenesis (Figure 1). Exosomes originate from the endosome and are the smallest EVs (30–150 nm in diameter). Microvesicles are larger (100–1000 nm in diameter) and are formed by the outward budding of the plasma membrane. Lastly, apoptotic bodies (50–5000 nm in diameter and usually in the large end of the scale) are released by dying cells [3,4]. However, differences in the techniques used to isolate EVs can make it hard to discriminate specific subpopulations, and consequently this review will not focus on specific subpopulations and collectively refer to all as EVs [5].

EVs facilitate cell-to-cell communication by delivering a specific cargo to recipient cells. The EV cargo is dynamic, and its content of nucleic acids, proteins, and lipids depends on the cell of origin and the status of that cell [5]. By delivering their cargos, EVs can promote or inhibit specific signaling pathways in the recipient cell and alter its phenotype, thereby playing an important role in disease development including NAFLD and progression to NASH. Whereas healthy hepatocytes produce EVs needed for cell survival and proliferation, stressed lipotoxic hepatocytes enhance the release of EVs that are able to promote disease progression by facilitating inflammation and fibrogenesis [7]. In this way, EVs contribute to hepatic inflammation via the recruitment of circulating immune cells and to hepatic fibrosis through the activation of hepatic stellate cells (HSCs), hereby promoting NASH progression [2]. The dynamic and varied cargos of EVs also suggest that they may act in different ways at different disease stages [6]. However, the role of EVs in cellular communication is intricate, and our understanding of EV function in NAFLD is rapidly changing. This review summarizes recent findings of EVs involvement in two of the major events in NAFLD progression: inflammation and fibrosis. We focus on the specific cargo mediating these effects in order to highlight potential therapeutic targets and potential disease biomarkers.

## 2. NASH Pathogenesis in Brief

The progression from a stage of bland steatosis to hepatic inflammation hallmarks the development of NASH. Lipotoxicity results in endoplasmic reticulum stress, lysosomal dysfunction, inflammasome activation, and cell death that collectively promotes the inflammation and infiltration of circulating immune cells [2]. The immunogenic environment of NASH is extremely complex and comprised of several cell types including monocytes, macrophages, neutrophils, natural killer cells, natural killer T cells, and T cells, infiltrating the liver and releasing a plethora of proinflammatory and -fibrogenic signaling molecules that promote disease progression and enhance the recruitment of additional immune cells in a self-sustaining feedforward loop [8]. Ultimately, chronic inflammation and injury signals activates HSCs which otherwise lie quiescent in the liver [9]. Activated HSCs are the primary cell type responsible for hepatic fibrosis and are characterized by increased proliferation and migration in addition to enhanced the production and deposition of extracellular matrices [9,10]. At the same time, they interact with infiltrating and resident immune cells as well as other hepatic cells to maintain a proinflammatory and -fibrogenic milieu [10]. Liver injury, including NAFLD, also results in the capillarization of liver sinusoidal endothelial cells (LSECs) with the ensuing loss of both fenestration and LSEC differentiation [11]. Concomitantly, LSECs become unable to suppress HSC activation, which further promotes fibrosis [12]. Ultimately, the development of hepatic fibrosis hallmarks a more serious stage of the disease associated with a substantial increase in mortality [13].

NAFLD is not restricted to the liver. Intercellular and interorgan communication is central to disease development and progression and to the association with several serious co-morbidities in humans such as type 2 diabetes, cardiovascular disease, and adipose tissue dysfunction [14,15,16]. Although, the liver seems to actively contribute to a reprogramming of distant cell populations and the promotion of disease development in other organ systems, the precise nature of the crosstalk between the affected organs is not fully understood [17]. A more in-depth knowledge of the cellular communications network involved in NAFLD progression not only constitutes an important research objective, but may also constitute an attractive therapeutic option, e.g., by manipulating specific networks, blocking progression and/or promoting the resolution of disease.

## 3. EVs as Mediators of NASH Progression

### 3.1. EVs Promote Inflammation

**Figure 2:**
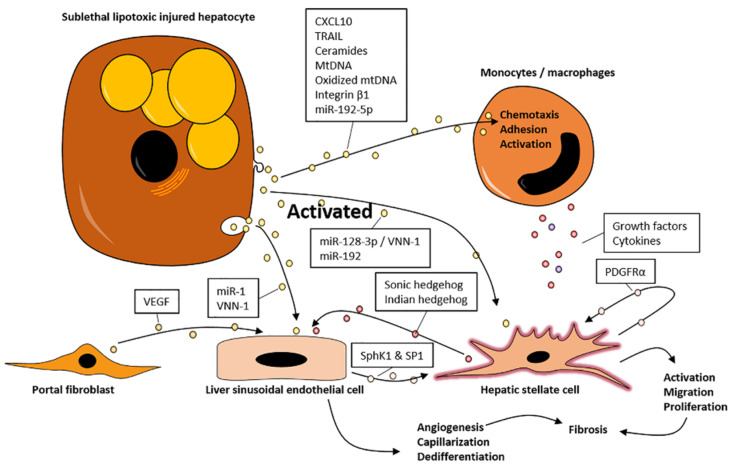
EVs as mediators of disease progression in NASH. EVs released by lipotoxic hepatocytes contain CXCL10, TRAIL, ceramides, mtDNA, oxidized mtDNA, integrin β1, and miR-192-5p that induce monocyte/macrophage chemotaxsis and promote a proinflammatory phenotype. Subsequently, activated immune cells release a plethora of cytokines and growth factors that activate hepatic stellates cells, thereby promoting fibrosis. Hepatic stellate cells may also be directly activated by miR-128-3p in VNN-1-expressing EVs and by EVs containing miR-192. Fibrosis is further facilitated by hepatocyte EVs carrying miR-1 and VNN-1 to liver sinusoidal endothelial cells resulting in angiogenesis, which is an important step in fibrosis. Likewise, portal fibroblasts may induce angiogenesis by releasing EVs containing VEGF. The hepatic stellate cells also actively contribute to disease progression via EV release, which reprogram recipient liver sinusoidal endothelial cells causing changes in their gene expression profile towards capillarization. Furthermore, liver sinusoidal endothelial cells may also promote hepatic stellate cell activation through EVs containing SphK1 and S1P, while the stellate cells themselves may initiate a self-sustaining signaling mechanism by releasing PDGFRα-enriched EVs. CXCL10: C-X-C motif chemokine ligand 10; mtDNA: mitrochondrial DNA; PDGFRα: platelet-derived growth factor receptor α. S1P: sphingosine-1-phosphate. SphK1: sphingosine kinase 1. TRAIL: tumor necrosis factor-related apoptosis-inducing ligand. VEGF: vascular endothelial growth factor. VNN1: vanin 1.

The chemotaxis, adhesion, and infiltration of circulating immune cells with subsequent establishment of a proinflammatory phenotype are crucial features in NASH, which is maintained by both tissue resident Kupffer cells (especially in early disease stages) and bone marrow-derived macrophages [17,18]. NAFLD research has consequently targeted the determination of factors that leads to the activation and recruitment of these immune cells, of which EVs are gaining increased attention [18]. The injection of circulating EVs isolated from high-fat-fed mice with NAFLD into chow-fed mice led to the hepatic accumulation of immature myeloid cells and increased levels of alanine and aspartate aminotransferase, linking EV signaling to alterations in hepatic health [19]. In contrast, EVs isolated from the chow-fed control mice did not elicit a similar response, implicating a proinflammatory role of EVs following the ingestion of an unhealthy diet [19]. This could be important in NASH, as patients are reported to ingest unhealthy diets high in fat and sugar, similar to diets used to induce hepatic steatosis in animal models [20]. Supporting a key role in inflammation, EVs are linked to immune cell chemotaxis. In vitro, lipotoxic hepatocytes increased EV production and released EVs containing C-X-C motif chemokine ligand 10 (CXCL10) through a mixed linage kinase 3 (MLK-3)-dependent mechanism [21]. These EVs increased the migration of bone marrow-derived macrophages in vitro, which could subsequently be blocked by CXCL10-neutralizing antisera. In vivo, CXCL10 knockout decreased hepatic macrophage infiltration in a murine model of diet-induced NAFLD [21]. Interestingly, the migratory induction by CXCL10 was more potent when packaged into EVs compared to the free chemokine, highlighting a role of EV signaling in the pathogenesis of NALFD [21] (Figure 2). Substantiating the clinical relevance of these findings, increased circulating levels of CXCL10 have been reported in NASH patients compared to in both patients with only steatosis and healthy controls [22]. Additionally, both MLK-3 and CXCL10 expressions were increased in the livers of NASH patients [21,22].

EVs may also contain bioactive lipid species (Figure 2). Lipotoxicity in cultured hepatocytes-induced stress in the endoplasmatic reticulum (ER) was mediated by inositol requiring enzyme 1α (IRE1α) and caused release of EVs enriched in C16:0 ceramide [23]. When added to murine bone marrow-derived macrophages in vitro, the ceramide metabolite sphingosine-1-phosphate (S1P) promoted macrophage migration, which could be blocked by sphingosine kinase (SphK) that catalyzes the formation of S1P inhibitors and S1P receptor inhibitors, supporting a link between lipotoxicity and macrophage recruitment in NAFLD [23]. The inhibition of S1P signaling reduced hepatic inflammation and fibrosis in a mouse model of NASH, further supporting the role of cytotoxic lipids in promoting the disease [24]. In humans, the ceramide content in EVs was reported to be higher in patients with steatosis or NASH compared to in obese controls, and ceramide concentrations were nominally higher in NASH patients compared to in patients with only steatosis [23]. IRE1α activation also increased ceramide synthesis, resulting in augmented EV production and increased hepatic macrophage accumulation in mice with NAFLD [25]. Moreover, the intravenous injection of these EVs enhanced hepatic macrophage accumulation in otherwise healthy mice [25]. Collectively, this suggests a mechanistic link between EVs released via IRE1α activation and subsequent ceramide synthesis and the hepatic infiltration of macrophages in NAFLD/NASH [25].

The progressing oxidative stress and lipid overload in NASH eventually lead to mitochondrial dysfunction and potential oxidative damage to the mitochondrial DNA [26,27]. EVs from obese patients with liver injury (elevated alanine aminotransferase) contained increased levels of oxidized mitochondrial DNA compared to from lean controls [28]. These EVs and the isolated mitochondrial DNA could activate toll-like receptor 9, which belongs to a family of receptors that are widely implicated in NASH [18,28]. Total or lysosome-expressing cell-specific knockout of toll-like receptor 9 (e.g., in Kupffer cells and infiltrating macrophages) protects against NASH, suggesting an additional connection between EVs and the progression of inflammation [28]. Following chemotaxis, the adhesion of arriving immune cells to LSECs constitutes a critical step in NASH-related liver inflammation, in which immune cells must pass through the fenestrated capillary wall to enter the hepatic parenchyma. EVs derived from in vitro cultured lipotoxic hepatocytes contained integrin β1, which has been shown to contribute to cell adhesion [29]. These lipotoxic hepatocyte-derived EVs appeared to enhance the adhesion of primary mouse monocytes to liver endothelial sinusoidal cells, hereby promoting the monocyte infiltration of the liver parenchyma [29]. Corroborating these findings, anti-integrin β1 treatment attenuated hepatic inflammation by decreasing monocyte trafficking to the liver in mice with diet-induced NASH [29].

In addition, EVs contribute to hepatic inflammation by inducing a proinflammatory phenotype in recipient cells (Figure 2). Accordingly, ER stress in cultured lipotoxic hepatocytes prompted the production of EVs through the ligand-independent activation of death receptor 5 (DR5) and rho-associated, coiled-coil-containing protein kinase 1 (ROCK1) pathways [30,31]. These EVs contained more than 2000 proteins including tumor necrosis factor-related apoptosis-inducing ligand (TRAIL), which in turn was able to activate DR5 possibly promoting further EV production [31]. The activation of DR5 on macrophages by TRAIL-containing EVs in vitro stimulated the NF-κB pathway to induce a proinflammatory phenotype characterized by the increased production of IL6 and IL1β [31]. Likewise, EVs isolated from NASH patients induced similar effects on macrophages in vitro [31]. These findings support the link between hepatocyte lipotoxicity and macrophage-mediated inflammation and suggest the inhibition of ROCK1-facilitated EV release as a therapeutic target in NASH [31].

In addition to lipids, cytokines, and oxidized molecules, EVs also transport a diverse range of noncoding cargos including miRNAs, which can alter gene transcription in recipient cells. In both patients and animal models of NASH, EVs contained increased amounts of miR-192-5p compared to in healthy controls and expressed markers (ASGPR1 and CYP2E1) consistent with a hepatocyte origin [32]. In vitro, EVs released by lipotoxic hepatocytes were taken up by macrophages and delivered miR-192-5p [32]. Subsequently, miR-192-5p promoted macrophage activation by signaling through Rictor (rapamycin-insensitive companion of mammalian target of rapamycin) to reduce the phosphorylation of Akt and FoxO1, ultimately resulting in the transcription of proinflammatory genes (*iNOS*, *IL6*, and *TNFa*) [32]. EVs released from lipotoxic hepatocytes were taken up by other hepatocytes and macrophages, leading to NLRP3 inflammasome activation and IL1β secretion in vitro [33]. Thus, lipotoxicity also sustains hepatic inflammation by facilitating the production of EVs that can reprogram hepatocytes and macrophages. As mentioned, the oxidative stress and oxidization of mitochondrial DNA play a role in the recruitment of inflammatory cells to the liver, but may also contribute directly to the activation of macrophages. Accordingly, the treatment of primary hepatocytes with H_2_O_2_ enhanced the production of EVs enriched with mitochondrial DNA that in turn induced the expression of inflammatory genes (*Tnfa*, *Il1b*, and *Il6*) in macrophages in vitro [34]. Notably, the activation of IL22 signaling altered EV cargos by decreasing the amount of mitochondrial DNA in vitro and in vivo suggesting that increased IL22 may be protective for NASH progression and potentially valuable as a therapeutic target [34]. In support, a phase 2a open-label study found that IL22 therapy (*n* = 18) was effective against alcoholic steatohepatitis [35]. Collectively, these results support a clear relationship between hepatocyte lipotoxicity and the subsequent development of hepatic inflammation. Importantly, a range of these studies have reported induced lipotoxicity without overt cell death, which is similar to the pathogenesis in vivo [21,23,31]. Thus, they underline a direct link between sublethal injury/stress induced by cytotoxic lipids in hepatocytes and the proinflammatory response as necessary for disease progression towards NASH.

### 3.2. EVs Promote Fibrosis

The risk of all-cause mortality and liver-related events increases with fibrosis progression (i.e., fibrosis stage) in patients with NAFLD [13]. Consequently, the development of fibrosis and the underlying mechanisms constitute a critical therapeutic target and endpoint in NAFLD research, with HSCs assumed as a pivotal role. Similar to inflammation, the links between lipid-laden hepatocytes and HSC activation remains poorly understood. EVs isolated from the plasma of high fat-fed NAFLD mice activated HSCs in vitro and increased mRNA levels of fibrosis-related genes *Col1a1*, *Col3a1*, *Mmp2*, and *Timp1* [36]. However, the EV cargo, which mediated this effect, was not characterized [36].

HSC activation is associated with decreased peroxisome proliferator-activated receptor γ (PPARγ) expression, while PPARγ agonists can reduce liver fibrosis in NASH patients [37]. miR-128-3p regulates PPARγ, and the expression of this miRNA was increased in the livers of both high fat- and choline-deficient amino acid-defined animal models of NAFLD [38]. Interestingly, lipotoxic hepatocytes released EVs containing increased levels of miR-128-3p, and these EVs suppressed PPARγ expression and promoted HSCs migration, proliferation, and activity in vitro [38]. The uptake of EVs was, partly, dependent on vanin-1 expression on the EV surface, with vanin-1-neutralizing antibodies leading to a decreased HSC activation in vitro and exemplifying the therapeutic potential of targeting EVs in the treatment of NASH [38] (Figure 2). The microarray analysis of EVs released by cultured lipotoxic hepatocytes identified 314 differentially regulated miRNAs compared to healthy hepatocytes [39]. In vitro, EVs from these lipotoxic hepatocytes increased the expression of the fibrogenic genes *ACTA2* (αSMA), *TGFB*, and *COL1A1* in HSCs, and this effect was, at least partially, mediated by miR-192 [39]. IL17 has been implicated in liver fibrosis, but the initial cellular origin and underlying signaling pathways are not yet fully elucidated, although EVs are likely to play a role [40]. Accordingly, EVs from CCl_4_-treated hepatocytes promoted CCL20 and IL17A production in HSCs by signaling through toll-like receptor 3 in vitro. In response to CCL20/IL17A, IL17A production was substantially enhanced in γδ T cells [40].

Angiogenesis is mediated by LSECs and correlates positively with the degree of liver fibrosis in patients with NASH [41,42]. Located in the space of Disse, LSECs are anatomically situated close to HSCs and may play a role in their activation, although it is currently unclear how this takes place. Human umbilical vascular endothelial cells exposed to EVs released from lipotoxic hepatocytes increased migration and tube formation in vitro [43]. Similarly, derived EVs also promoted angiogenesis in vivo in mice [43]. In contrast, EVs isolated from mice fed a high fat and high carbohydrate diet did not induce angiogenesis in vitro [43]. However, mice in the latter study developed less severe NASH, indicating that proangiogenic EVs are only produced at later disease stages, at least in mice. Nevertheless, the angiogenic effects of the EVs were found to be dependent on EV internalization mediated by vanin-1 [43] (Figure 2). In conjunction with the role of vanin-1-positive EVs in HSC activation, these results support that vanin-1-positive EVs may be explored as therapeutic targets in NASH [38,43]. Clonally-derived rat HSCs activated by platelet-derived growth factor (PDGF)-BB in vitro produced EVs containing both sonic and indian hedgehog and induced gene expression changes associated with capillarization and nitric oxide in primary LSECs, thereby potentially contributing to the vascular changes associated with liver fibrosis [44]. Cytotoxic lipids transported by EVs also affect fibrogenesis. Similarly, circulating EVs containing increased levels of SphK1 and SP1 were found in mice with CCl_4_-induced liver fibrosis [45]. EVs from SphK1-overexpressing LESCs contained increased levels of SphK1 and SP1 and induced HSC migration in vitro [45]. This accentuates that EVs can function as mediators of lipotoxicity and that they are able to transfer harmful lipid species to recipient cell populations, leading to changes in expression patterns and phenotypes supporting EVs as central in the LSEC–HSC communication network. In healthy primary hepatocytes, EVs deliver SphK2, which increases S1P synthesis in recipient hepatocytes and promotes proliferation and liver regeneration following ischemia/reperfusion injury or partial hepatectomy [46]. This further supports the EVs role as dynamic vehicles of signal transfer between cell populations in the liver and implies SphK-/S1P signaling to be context-dependent [46].

The communication between vasculature and myofibroblasts (such as HSCs) is not unidirectional as a portal myofibroblasts signal to endothelial cells and promotes angiogenesis via EVs containing vascular endothelial growth factor A both in vivo and in vitro [47]. Furthermore, the expression of the portal myofibroblast marker *COL15A1* was increased in liver samples from patients with NASH and advanced fibrosis (bridging/cirrhosis), but not in patients with mild–moderate fibrosis or bland steatosis compared to in healthy controls [47]. *COL15A1* expression correlated with the endothelial marker von Willebrand factor, suggesting a link between fibrosis and angiogenesis [47]. Thus, EV-mediated angiogenesis may be an important contributor to the fibrogenesis from the portal areas in NASH (Figure 2). Still, fibrosis initially has a centrivenular origin in adult NASH, and the vast majority of myofibroblasts are derived from HSCs in NAFLD. HSCs rather than portal myofibroblats may, therefore, be a more relevant target for future antifibrotic treatments in NASH [9,48]. HSCs are not only affected by EVs released by other cells, but also release EVs that serve in a paracrine manner to activate additional HSCs and promote fibrosis. In vitro, EVs released by activated HSCs contained 337 different proteins associated with extracellular spaces or matrices and collagens, whereas quiescent HSCs produced EVs containing only 46 proteins that mainly associated with histones and keratins [49]. EVs extracted from activated HSCs enhanced the expression of fibrogenic genes (connective tissue growth factor (*Ctgf/Ccn2*), *Col1a1*, and *Acta2* (αSMA) in quiescent HSCs in vitro. Conversely, EVs from quiescent HSCs decrease fibrogenic gene expression in activated HSCs [49]. Cultured quiescent HSCs have also been shown to produce twist-related protein 1-containg EVs in turn promoting miR-214 expression [50]. These EVs inhibited the expression of CTGF in recipient HSCs, thereby ablating fibrogenic signaling [50]. Conversely, miR-214 and twist-related protein 1 levels were much lower in EVs isolated from activated HSCs in vitro, which could make these EVs less effective for suppressing fibrogenic signaling [50]. Moreover, levels of miR-214 and miR-199a-5p were increased in EVs from quiescent HSCs compared to from activated HSCs in vitro [51,52]. EVs released by quiescent HSCs and subsequently internalized by activated HSCs decrease the expression of markers of activation/fibrogenesis (CTGF/CCN2, COL1A1, and ACTA2 (αSMA)) in vitro [51,52]. Hence, EV-transported miRs seems to be important factors in the regulation of the HSC phenotype and hepatic fibrosis.

PDGF induces HSC proliferation and migration by binding to PDGF receptors [53]. PDGF plays a central role in NASH and is secreted by several cell types implicated in disease development and progression, including Kupffer cells, monocyte-derived macrophages, and biliary epithelial cells [53]. PDGF receptor α was enriched in circulating EVs isolated from cirrhotic patients with alcoholic-related liver disease, and in vitro cultured HSCs treated with PDGF-BB release PDGFRα-enriched EVs in a Src homology 2 domain tyrosine phosphatase 2 (SHP2)-dependent manner [54]. These EVs promoted migration of cultured HSCs and enhanced liver fibrosis when administrated to CCl_4_-treated or bile duct-ligated mice [54]. Subsequent inhibition of SHP2 ameliorated fibrosis [54]. Mechanistically, SHP2 induced mTOR signaling, in turn inhibiting HSC autophagy and promoting the release of profibrogenic EVs [54]. This highlights an important role of autophagy in HSC-mediated liver fibrosis [55]. Together, these results illustrate a role of HSCs in the paracrine signaling associated with hepatic fibrosis and suggest that the activation of HSCs leads to qualitative and quantitative changes in their EV cargos, which can alter other HSCs and drive fibrosis progression. Conversely, an altered EV cargo may reduce HSC activation and inhibit profibrotic signaling.

## 4. EVs May Promote NASH via Organ Crosstalk

The detrimental effects of NASH are interlinked with other organ systems between which considerable crosstalk occurs. Accordingly, NASH is not only associated with increased risk of liver-related mortality, but also with cardiovascular death, type 2 diabetes mellitus, and chronic kidney disease [56]. However, the interplay between the liver and other organs in NAFL and NASH remains poorly understood. Recent reports suggest the liver as central in altering expression patterns in distant organs in response to lipid overload [57]. In mice, high-fat feeding leads to an accumulation of lipids in the liver prior to the accumulation in adipose tissue [57]. The increase in intrahepatic lipids leads to a geranylgeranyl diphosphate synthase (Ggpps)-dependent secretion of hepatocyte-derived EVs that enhanced lipid accumulation in preadipocytes in vitro [57]. This effect was mediated, at least in part, by the miRNA let-7e-5p, which enhanced adipocyte lipogenesis while decreasing fatty acid oxidation and increasing lipid accumulation. Furthermore, the adipose tissue fat mass decreased significantly in high fat-fed mice with liver-specific *Ggpps* knockout [57]. This seminal study emphasizes a role of the liver–adipose tissue axis and organ-to-organ signaling during NAFLD as well as a crucial role of hepatocyte-derived EVs in promoting metabolic adaptation in adipose tissue. Furthermore, EVs released by human subcutaneous and omental adipose tissue ex vivo inhibited insulin-mediated Akt phosphorylation in hepatocytes in vitro, suggesting the existence of a bidirectional communication between adipose cells and hepatocytes [58]. Cultured lipotoxic hepatocytes released EVs containing more than 500 differentially regulated miRNAs with a marked upregulation of miR-1. Subsequent in vitro and in vivo studies found that these EVs facilitate the crosstalk between the liver and vascular endothelium in NAFLD [59]. The EVs from lipotoxic hepatocytes delivered miR-1 to endothelial cells leading to endothelial inflammation and atherosclerosis, and the inhibition of miR-1 decreased the inflammation and the size of atherosclerotic lesion in high fat-fed *ApoE^−/−^* mice, directly linking NAFLD-induced lipotoxicity to cardiovascular disease through the composition of EV cargos [59].

## 5. EVs as Biomarkers in Patients with NASH

At present, the diagnosis of NASH relies almost exclusively on histopathological features assessed in liver biopsies. The procedure is costly, invasive and prone to sample variability, thereby constituting a major limiting factor in NASH research [60]. Although other diagnostic tools such as imaging techniques and serum markers are available and show promise, they are not unequivocally associated with disease progression, and there is a clear and urgent need to develop additional noninvasive procedures for accurate NASH diagnosis and longitudinal monitoring of disease development [61,62]. Since EVs are released to the circulation, they may well constitute an attractive option for a noninvasive diagnostic marker (Table 1).

Compared to in healthy controls (*n* = 25), the number of circulating EVs was increased in NASH patients with cirrhosis (*n* = 25, F4) and also nominally increased in precirrhotic (*n* = 25, F3) NASH patients [63]. Hepatocyte-specific EVs (ASGPR1- or SLC27A5-positive) accounted for 20% of the circulating EVs and ASPGR1-positive EVs correlated with fibrosis stage, NAFLD fibrosis score, and the enhanced liver fibrosis (ELF) score (designed to diagnose severe fibrosis (grades 3 and 4)). In addition, the total number of hepatocyte EVs could identify clinically relevant portal hypertension in cirrhotic patients (AUROC = 0.79). Finally, the authors identified proteomic signatures in EVs that enabled a differentiation between advanced NASH (pooled F3 and F4) and healthy controls (AUROC = 0.77) and between precirrhotic and cirrhotic NASH (AUROC = 0.80) [63]. This supports that the protein cargo of circulating EVs may be specifically related to the disease stage and may therefore be used to diagnose and stage patients with NASH. Kornek et al. analyzed circulating EVs for six different cell surface markers belonging to major immune cell populations involved in liver inflammation and fibrosis [64]. The number of circulating EVs from invariant natural killer cells and CD14^+^ monocytes/macrophages differentiated patients with NAFLD (*n* = 67) from patients with hepatitis C (*n* = 42) and healthy controls (*n* = 44) and may represent a novel diagnostic tool for not only separating NAFLD patients from healthy individuals, but also differentiating between various chronic liver diseases [64]. In patients with NAFLD and advanced fibrosis (*n* = 9, grades 3 and 4), the numbers of circulating leucocyte and endothelial cell EVs were decreased compared to in NAFLD with no/mild fibrosis (*n* = 17, grades 0–2) [65]. Furthermore, adding either CD14^+^ or CD16^+^ EVs to the ELF score improved its diagnostic potential [65]. In plasma from patients with alcohol/hepatitis C virus-related cirrhosis (*n* = 91) compared to in healthy controls (*n* = 30), EVs from leuko-endothelial (CD31^+^/CD41^−^), lymphocyte (CD4^+^), and erythrocyte (CD235a^+^) were increased, and EV-bound cytokeratin-18 correlated positively with liver disease activity (Child-Pugh score and Model for End-Stage Liver Disease (MELD) score) [69]. Likewise, circulating cytokeratin-18 levels (not EV-associated) were also found to be increased in patients with NASH (*n* = 41) compared to in patients without NASH (*n* = 54) and could be applied diagnostically (AUROC = 0.86) [70]. Constituting another potential biomarker, EV-associated integrin β1 expression was shown to promote disease progression by facilitating monocyte recruitment, and these EVs were also increased in patients with NASH and mild (F1–2) fibrosis compared to in patients with steatosis [29].

In accordance with the central role of lipids in NAFLD, C16 ceramide and S1P concentrations were increased in EVs isolated from patients with steatosis and even further in patients with NASH and none/mild (F0–1) fibrosis compared to in obese controls [23]. Mitochondrial DNA was also increased in EVs from obese patients with elevated alanine aminotransferase levels compared to from lean controls [28]. However, aminotransferase levels poorly predict NAFLD/NASH, and additional studies are needed to investigate the potential of EVs expressing mitochondrial DNA as markers in patients with biopsy-confirmed disease. EV-associated miRNA levels may also be useful in identifying patients with NASH. A small study reported higher levels of miRNA-122 and -129 in advanced (*n* = 3) compared to in early (*n* = 3) NAFLD [39]. EVs from patients with NAFLD/NASH (*n* = 34) contained higher levels of miRNA-16, -34a, and -122 compared to from healthy controls (*n* = 19), and EV miRNA-16 and -122 could differentiate between NASH and controls (AUROC = 0.96 and 0.93, respectively) [68]. Likewise, miRNA-122, -192, and -375 were enriched in EVs from patients with NASH (*n* = 47) compared to from those with steatosis (*n* = 30) or healthy controls (*n* = 19), and EV miRNA-122 could, to some degree, predict NASH (AUROC = 0.71) and fibrosis (AUROC = 0.61) [66]. EV miR-192-5p levels were higher in NASH patients (*n* = 31) compared to in healthy controls (*n* = 37) and could be investigated further as a NASH biomarker [32]. The microarray analysis of “exosome rich fractionated RNA” from patients with NASH (*n* = 12), hepatitis B (*n* = 4), and controls (*n* = 24) identified a panel of 12 miRNAs, which could differentiate NASH from both controls and hepatitis B patients [67].

## 6. Conclusions

EVs released into the local hepatic environment and to the systemic circulation may directly contribute to the development and progression of NASH. Central to the production of these EVs are lipotoxic hepatocytes, and the EVs released by these cells provide a tangible link between the initial lipid accumulation in NAFLD and the subsequent development of hepatic inflammation and fibrosis. Why some cells succumb to the detrimental effects of lipotoxicity and the initiate production of EVs is a target for future investigations. Likewise, the susceptibility of recipient cells of uptake of lipotoxic EVs may also constitute an important checkpoint in disease progression. However, the role of EVs as signal carriers appears central in facilitating disease progression and reprogramming of cell populations in NAFLD/NASH.

## Figures and Tables

**Table 1:** Cargos of circulating EVs as biomarkers in patients diagnosed with NAFLD.

Study Design & NAFLD Diagnosis	Cellular Source	EV Cargo
Cirrhotic NASH (*n* = 25, F4), pre-cirrhotic NASH (*n* = 25, F3) and healthy control (*n* = 25). Biopsy [63]	Total circulating and hepatocyte (ASGPR1- or SLC27A5-positive)	Proteomic signature of circulating EVs differentiates advanced NASH (F3 + F4) from healthy controls (AUROC = 0.77) and precirrhotic from cirrhotic NASH (AUROC = 0.80)
NAFLD (*n* = 67) vs. HCV patients (*n* = 42) or healthy controls (*n* = 44). Biopsy [64]	iNKT (Vα24/Vα11 positiv) or macrophages/monocytes (CD14^+^)	Number of iKT EVs to differentiate NAFLD from controls (AUROC= 0.92) and HCV (AUROC = 0.97) Number of CD14^+^ EVs differentiate NAFLD from controls (AUROC = 0.83) and HCV (AUROC > 0.99)
Advanced NAFLD, fibrosis 3 and 4 (*n* = 9) vs. early NAFLD, fibrosis 0–2 (*n* = 17). Biopsy [65]	Leukocytes (CD14^+^ or CD16^+^) Endothelial cells (either CD105^+^ CD31^+^ CD41/CD42^−^, CD105^+^ CD31^−^ CD41/CD42^−^, or CD105^−^ CD31^+^ CD41/CD42^−^)	↓ Number of leucocyte and endothelial cell EVs in advanced NAFLD
NASH with mild (F1–2) fibrosis (*n* = 17) vs. steatosis (*n* = 8). Biopsy [29]	Not examined	↑ Integrin β1 in NASH
NASH F0–1 fibrosis (*n* = 16) vs. bland steatosis (*n* = 16) or obese controls (*n* = 11). Biopsy for some [23]	Not examined	↑ C16:0 ceramides and S1P in bland steatosis and NASH. Nominally increased in NASH vs. bland steatosis
Obese/high ALT (*n* = 9) vs. obese/normal ALT (*n* = 19) or lean/normal ALT (*n* = 19). Elevated ALT [28]	Hepatocyte (ARG1 positive, CD41 negative)	↑ mtDNA in obese with high ALT
NASH (*n* = 47), steatosis (*n* = 30) and health controls (*n* = 19). Biopsy [66]	Not examined	↑ miRNA-122, -192 and -375 in NASH vs. steatosis or healthy controls miRNA-122 could to a degree identify NASH (AUROC = 0.71) and fibrosis (AUROC = 0.61)
Advanced NAFLD (*n* = 3) vs. early NALFD (*n* = 3). Biopsy [39] ^†^	Not examined	↑ miRNA-122 and -192 in advanced NAFLD
NASH (*n* = 31) vs. healthy controls (*n* = 37). Biopsy [32]	Hepatocyte (ASPPR1 and CYP2E1 positive)	↑ miR-192-5p in NASH
NASH (*n* = 12), hepatitis B (*n* = 4) and controls (*n* = 24). Biopsy [67]	Not examined	miRNA panel (miR-1225-5p, -1275, -368, -762, 320c, -451, -1974, -630, -1207-5p, -720, -1246, and -486-5p) distinguish NASH from HBV and controls with accuracies of 87.5% and 88.9%, respectively
NAFLD/NASH (*n* = 34) vs. healthy controls (*n* = 19). Biopsy [68]	Not examined	↑ miRNA-16, -34a, and -122 in NAFLD/NASH miRNA-16 (AUROC = 0.96) and miRNA-122 (AUROC = 0.93) differentiates NAFLD from healthy controls

^†^ Early NALFD = grade 1 steatosis, grades 0–1 fibrosis. Advanced NAFLD = grade 2 steatosis, grades 2–3 fibrosis [39]. ALT: alanine aminotransferase; AUROC: Area under the receiver operating characteristics curve EVs: extracellular vesciles; iNKT: invariant natural killer T cells; NAFLD: nonalcoholic fatty liver disease; NASH: nonalcoholic steatohepatitis. Up and down arrows: Increased and decreased markers, respectively.

## Data Availability

No new data were created or analyzed in this study. Data sharing is not applicable to this article.
